# Management of Leishmaniases in the Era of Climate Change in Morocco

**DOI:** 10.3390/ijerph15071542

**Published:** 2018-07-20

**Authors:** Kahime Kholoud, Sereno Denis, Bounoua Lahouari, Moulay Abdelmonaim El Hidan, Bouhout Souad

**Affiliations:** 1 Laboratory of Applied Sciences for the Environment and Sustainable Development, School of Technology -Essaouira, Cadi Ayyad University, Marrakesh 40000, Morocco; kahimkholoud@gmail.com; 2 IRD, University of Montpellier, InterTryp, 34000 Montpellier, France; 3 IRD, University of Montpellier MiVeGec, 34000 Montpellier, France; 4 Biospheric Sciences Laboratory, NASA’s Goddard Space Flight Center, Greenbelt, Maryland, MD 21401, USA; lahouari.bounoua-1@nasa.gov; 5 Science and Technology Center—Ait Melloul University Campus, Ibn Zohr University, Agadir 80000, Morocco; elhidan@gmail.com; 6 Directorate of Epidemiology and Disease Control, Ministry of Health, Rabat 10010, Morocco; souadbouhout@gmail.com

**Keywords:** insect-borne diseases, leishmaniases, climate change, Morocco

## Abstract

The proliferation of vector-borne diseases are predicted to increase in a changing climate and Leishmaniases, as a vector-borne diseases, are re-emerging diseases in several regions of the world. In Morocco, during the last decade, a sharp increase in cutaneous leishmaniases cases has been reported. Nevertheless, in Morocco, leishmaniases are a major public health problem, and little interest was given to climate change impacts on the distribution and spread of these diseases. As insect-borne diseases, the incidence and distribution of leishmaniases are influenced by environmental changes, but also by several socio-economic and cultural factors. From a biological point of view, environmental variables have effects on the survival of insect vectors and mammalian reservoirs, which, in turn, affects transmission. Here, we highlight the effects of climate change in Morocco and discuss its consequences on the epidemiology of leishmaniases to identify challenges and define targeted recommendations to fight this disease.

## 1. Introduction

Leishmaniases are vector-borne diseases caused by obligate parasites from the genus *Leishmania* (*Trypanosomatida*: *Trypanosomatidae*), transmitted by the bites of an infected female Phlebotomine sand flies (Diptera: Phlebotominae) and hosted by a wide variety of mammals, playing the role of reservoir [1,2,3]. They are mandatorily reportable diseases in Morocco (Ministerial Order No. 683-95 of 31 March 1995). In Morocco, various *Leishmania* species are endemic: (i) *Leishmania infantum* is the causative agent of both visceral (VL) and sporadic cutaneous leishmanisis (CL) infection; (ii) *L. tropica* is responsible for the anthroponotic cutaneous leishmaniasis (ACL); and (iii) *L. major* is the causative agent of the zoonotic cutaneous leishmaniasis (ZCL). In Morocco, three species of phlebotomine sandflies are currently proven or suspected vectors of *L. infantum*: *Phlebotomus ariasi*, *P. perniciosus*, and *P. longicuspis* [1,4]. *P. papatasi* and *P. sergenti* are the proven vectors of *Leishmania major* and *L. tropica*, respectively, in the country [1,3,5,6]. The identified reservoir hosts are dogs for zoonotic VL, rodents for ZCL, and humans for ACL [3,7].

The Fifth Assessment Report (AR5) of the Intergovernmental Panel on Climate Change (IPCC) highlighted the exponential increase in the number of publications documenting climate change and its impact on health [8]. Among them, leishmaniases are significantly affected by the change and instability of the climate [9]. As a vector-borne disease, the impact of climate change on the incidence and distribution of leishmaniases is well documented [3,10,11,12,13,14,15]. In Morocco, the visceral form of the disease is endemic in the Rift and pre-Rift mountains with over 150 cases per year reported from 2006 to 2008 [3,4]. Cutaneous leishmaniasis is caused by *L. major* or *L. tropica*; CL was previously sporadic and became epidemic in 1976. The disease occurs in unpredictable outbreaks in the south and the southeast regions of the Atlas Mountains and recently migrated from the west to the east of the country. In 2001, the Moroccan Health Office reported 2028 CL cases caused by *L. major* and *L. tropica* and 3414 cases in 2008, showing a sharp increase in the incidence of leishmaniases in Morocco. Recent data confirm that, in Morocco, leishmaniases has spread to new areas in line with a significant increase in the total number of recorded cases [16,17]

This change can, in part, be attributed to a low efficiency of the leishmaniasis control program. Nevertheless, bioclimatic, soil, vegetation, and climate change, as well as other socioeconomic factors, may also contribute to the dynamic and geographic expansions of these diseases see Table 1 [12,13,17,18]. To address changes in their preferred bioclimatic niches driven by climate change, Phlebotomine vectors, as well as mammalian reservoirs, can spread to new more suitably available habitats. However, these movements are limited by their own intrinsic biological resilience and the potential of these vectors and reservoirs [19]. In Iran, models using degree/day highlighted a link between the climatic variable and the dynamics of activity of the main vector of VL in this country [20]. A high level of precipitation and vegetative foliage are favorable habitats for both reservoirs and vectors of cutaneous leishmaniasis [11,13]. In addition, climate change may also have effects on the transmission, via the socio-economic change they produce, i.e., the impact on the amount of human contact with the transmission [4,21]. This opinion paper will focus on the impact of climate change on the epidemiology of leishmaniases in Morocco and will discuss methods to turn this knowledge into intervention.

## 2. Climate Change in Morocco

The Fifth Assessment Report of the IPCC [8] indicates that warming of the global climate system is both unequivocal and evidenced by observations of increases in the global mean air and ocean temperatures, melting of ice, and rising of the average sea level. In the North African region, including Morocco, the IPCC models unanimously agree that general warming has demonstrably occurred [8]. Morocco is located between the arid zones of the Sahara and the moderate Mediterranean and Atlantic regions. According to the Second National Communication [22], future climate prediction may favor an expansion of the aridity to the north of the country. The analysis of temperature evolution between 1960 and 2000 highlights an increase of a maximum of 1.4 °C in the average temperature during this period, as well as an upward trend in both minimum and maximum temperatures. In Morocco, the predicted consequences are an increase in the overall temperature associated with a reduction in precipitation, leading to a northward expansion of arid climate characteristics [23]. Morocco is considered a country vulnerable to the adverse impacts of global climatic change [8]. The main reasons raised are inadequate adaptive capacity for high water stress, socio-ecological and economic problems linked to food and water security, and uncontrolled migration.

## 3. Leishmaniases Epidemiology in Morocco: What Is Known and What Can We Anticipate from Climate Change 

**Figure 1:**
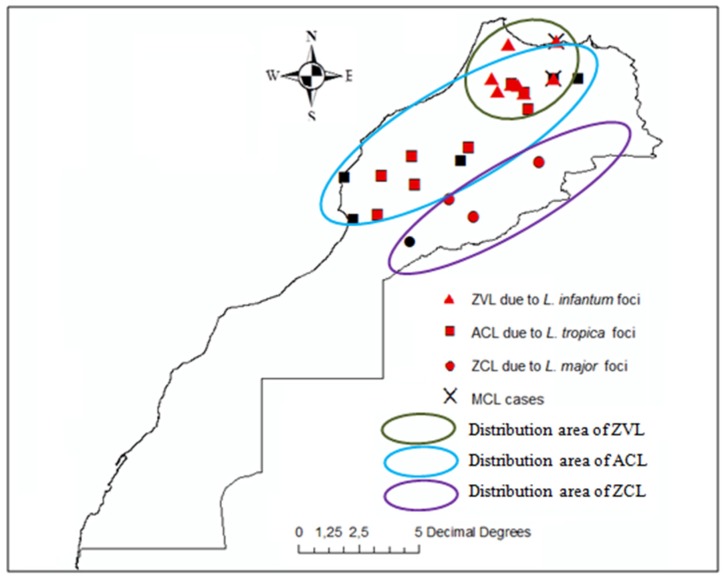
Geographical distribution of leishmaniases clinical forms in Morocco. Zoonotic visceral leishmaniasis (ZVL) Anthoponotic cutaneous leishmaniasis (ACL) zoonotic cutaneous leishmaniasis (ZCL) Mucocutaneous leishmaniasis (MCL).

Leishmaniasis, a neglected tropical disease, constitutes a major public health threat to the Moroccan nation (Figure 1). Specifically, it severely impedes economic development by affecting poor rural populations [24]. The cutaneous forms of the disease represent a major socio-economic constraint among women and children by curtailing, through aesthetic disfigurement, the possibility of normal interpersonal relations, whereas the visceral form, which mainly affects children, is fatal if untreated [25]. Over the past decade, the analysis of epidemiological data has highlighted a geographic expansion to previously leishmaniasis-free areas in conjunction with the emergence of new foci in several provinces of Morocco [26]. In terms of the total number of registered leishmaniases cases, 24,804 cases due to *L. major* CL, 16,852 cases due to *L. tropica* CL and 1366 cases caused by *L. infantum* VL were recorded between 2004 and 2013 in Morocco [16,26]. For the 1990–1999 period, the Moroccan Ministry of Health reported 10,366 cases of *L. major* CL, 488 cases of *L. tropica* CL, and 539 cases of *L. infantum* VL [27]. In terms of geographic distribution, a migration of *L. major* (ZCL) cases from the southeast to the northeast of Morocco was observed. Cases of cutaneous lesions caused by *L. tropica* (ACL) became more common and overlapped with foci of *L. infantum* (VL) [28]. Moreover, and unexpectedly, in Southern Morocco, visceral leishmaniasis caused by *L. infantum* appeared more frequently [14]. In Morocco, malaria was eradicated after 2004. Since then, the Ministry of Health halted the fight against *Anopheles* (the insect vector of the malaria parasite) via dichloro-diphenyl-trichloroethane (DDT) use. The absence of such previously common DDT use may favor sand fly proliferation; however, climate change should also be suspected of being a driver of disease spread [13,29].

The impact of climate change, particularly changes in temperature, affects the distribution of leishmaniases via sand fly abundance or via the effect of temperature on the parasite development process as its vector has been stressed as early as 2008 [21]. The Phlebotomine sand fly distribution is limited to areas with temperatures above 15.6 °C for at least three months of the year [30]. Below 10 °C, sand flies must enter a dormant state to survive winter [31]. Experimental laboratory data demonstrated that ambient temperature significantly impacts various metabolic and digestive processes that affect the developmental life cycle of sand flies [32]. Nevertheless, if ambient temperature directly affects sand fly development, its impact on the expansion range in natura is less clear because of the likely influence of the photoperiod on the overwintering diapause that acts as a confounding cofactor [33]. Bounoua et al., (2013) proposed that the increase in ZCL cases reported is related to an increase in the minimum temperature, which allowed sand fly larvae to survive winters, thereby creating conditions suitable for an endemicity that did not previously exist [13]. The impact of climate change on parasite development within its vector has received little attention, although it is demonstrated that *L. infantum* develops better at higher temperatures in the digestive tract of *P. ariasi* [34], and this was further investigated in other *Leishmania* species [29]. *Leishmania* parasites developed faster at higher temperatures only during the early stages of sand fly gut colonization [29]. If the transmission of *Leishmania* by sand flies is sensitive to temperature change, many other factors may also play a role in the modulation of its spread. Of these, some are linked to the host (immune, nutritional or genetic status of the host) [35], the parasite (virulence and drug resistance) [36], and socio-economical determinants (travel and migration of human populations, urbanization, demographic, lifestyle, and availability of health services) [3,24,25]. Regarding cutaneous leishmaniases, social stigma was shown to reinforce poverty in affected individuals and, thus, is of great concern [25]. Moreover, there is a continuation of psychological morbidity because lesion visibility is an important risk factor for depression in dermatological conditions [37]. In Southeast Morocco, women suffer from the long-term stigmatizing effects of inactive CL scarring, creating a feeling of shame, and this suggests that the disease is a large-scale social health problem [24]. Therefore, this is a real social problem, and women affected need psychological monitoring as well as awareness campaigns. These campaigns are required to explain the global context of disease transmission and its cycle, symptoms, treatment, and prophylaxis.

Beyond increased awareness, there is an urgent need for more strategic thinking about vector/reservoir control that could reduce the risk of transmission of leishmaniases (area, period, and population) that may be increased by climate change and/or other environmental factors. Although there remain uncertainties about climate change models, climate change is likely to cause an increase in the prevalence, distribution, and severity of leishmaniases in Morocco.

## 4. Managing Leishmaniases in Morocco in the Era of Climate Change: A Puzzling Problem

The National Ministry of Health of Morocco set up basic intervention strategies for the prevention and control of leishmaniases to reduce the incidence of cutaneous leishmaniasis to 50% by 2021 and to avoid mortality related to visceral leishmaniasis. To reach this goal, the interventions will primarily focus not only on the early screening of leishmaniases and case management, but also on strengthening the prevention and control actions within the framework of vector control. The vector control strategy should be reassessed and implemented in a framework of decision-making and quality assurance that can be applied at the lowest administrative system [38,39]. Another aspect is to pursue the control of rodents and vectors through physical and/or chemicals methods. To face the consequences of climate change on insect-borne disease incidence and spread, emphasis should be placed on the inclusion of climate change risks and impacts in the national health strategy. The impact of climate change on insect-borne diseases, such as leishmaniases, is currently ongoing and requires stronger and earlier biological-based evidence. In Morocco, the lack of recent entomological data resulting from the long-term monitoring of sand fly diversity and density, as confirmed through sampling along some well-identified transects, is currently challenging. These data will help the future delineation of the altitudinal and latitudinal distribution of sand fly species that play an important role in *Leishmania* transmission. In addition to data on the diversity, data about the density and distribution of potential leishmania reservoirs are also lacking. These data are crucial to generating risk maps to define the spatial and temporal vulnerability, as well as the dispersion and colonization of vectors and reservoirs. Additionally, as recommended by Courtenay et al. (2017), a better understanding of the relationships between sand flies and mammalian hosts are needed to improve knowledge about transmission, the observed epidemiology of the disease, and their implications in the choice of control strategy [40].

In addition to these bio-ecological data, it is also important to assess the economic and socio-cultural risks, prioritized by experts, and to associate these with scenarios that use various climate parameters, including land use/cover, poverty, urbanization, migration, and demography trends. 

Therefore, effort should be given to selecting adequate indicators, in terms of time and space, to gather data with appropriate quality in terms of resolution, units, sensitivity, specificity, and accuracy. This will help define and establish an effective prevention strategy consistent with events previously defined (risk maps, monitoring, and health rules) and categorized by the region and period at risk. There are several examples (a) at a microscale: hygiene and animal management, (b) at a mesoscale: irrigation systems and oasis areas; and (c) at a macroscale: population migrations and droughts. Finally, it is necessary to develop and adopt an early warning system based on the integration of statistical/spatial prediction models using monthly or yearly climatic and disease outbreak data, density or activity for both vectors and reservoirs, and an appropriate field-based surveillance response, which will enhance surveillance.

## Figures and Tables

**Table 1:** Factors that play a role in leishmaniasis, cutaneous leishmaniasis (CL), expansion.

Foci	CLinical Presentation	Leishmania Cycle	Underlying Causes of Expansion	References
Governorate of Sidi Bouzid, central Tunisia	Zoonotic Cutaneous Leishmaniasis	Species: *Leishmania major* Reservoirs: *Psammomys obesus*, *Meriones shawi and Meriones libycus* Vector: *Phlebotomus papatasi*	- Higher rainfall is expected to result in increased densities of chenopods and rodents - Emergence of ZCL epidemics can take place when human socio-economic activities interfere with the ecologic niche of reservoirs	[12]
Village of El M’hir, North of Algeria	Zoonotic Cutaneous Leishmaniasis	Species: *Leishmania major* Reservoirs: *Psammomys obesus* Vector: *Phlebotomus papatasi*	- Desertification observed in the steppe area northern Sahara	[18]
Errachidia province, South-Est of Morocco	Zoonotic Cutaneous Leishmaniasis	Species: *Leishmania major* Reservoirs: *Meriones shawi* Vector: *Phlebotomus papatasi*	- Trophic cascade in leishmaniasis complex. Increase rainfall resulting in an increased densities of chenopods and rodents - Increase in minimal temperature Impacts the *P. papatasi* activity - Bioclimatic, vegetqtion qnd soil - Socioeconomic factors	[13]
Old world	Cutaneous Leishmaniasis	Species: *L. infantum, L. tropica, L. major*, *L. aethiopica and L. donovani* Reservoirs: Humans, rodents, Canids, Hyraxes, … Vector: *Phlebotomus papatasi, P. sergenti*, *P. caucasicus, P. duboscqi*, *P. Bergeroti, …*	- Environnental change, climate change, urbanisation, socioeconomic factors, human behavior, malnutrition, Population mouvement…	[17]
